# Cardiac tamponade complicating thoracentesis in a patient after left pneumonectomy: A case report

**DOI:** 10.1097/MD.0000000000019778

**Published:** 2020-04-10

**Authors:** Mingxia Zheng, Yu Kang, Tong Wang, Jiafu Wei

**Affiliations:** Department of Cardiology, West China Hospital, Sichuan University, Chengdu, Sichuan, China.

**Keywords:** cardiac tamponade, complication, thoracentesis

## Abstract

**Rationale:**

Therapeutic or diagnostic thoracentesis is widely used in different clinical settings. Cardiac injury, a rare complication, could lead to fatal consequences. We describe a case of cardiac tamponade complicating thoracentesis that was recognized and rescued in a timely manner.

**Patient concerns:**

A 42-year-old woman underwent blind thoracentesis due to excessive left pleural effusion after left pneumonectomy surgery. She suddenly lost consciousness and was in a state of shock a few minutes after needle insertion and fluid drainage.

**Diagnosis:**

Bedside transthoracic echocardiography revealed pericardial effusion at a depth of 20 mm, and cardiac tamponade complicating thoracentesis was diagnosed.

**Interventions:**

After draining 250 mL of non-coagulated blood by pericardiocentesis under transthoracic echocardiography guidance, a tube was placed for continuous drainage over the subsequent 36 hours.

**Outcomes:**

The patient's hemodynamic condition was stabilized hours after pericardiocentesis. The patient was discharged in good condition a few days later.

**Lessons:**

Imaging assessment and guidance in the process of thoracentesis was indispensable, especially in a patient with altered intra-thoracic anatomy. Cardiac damage, as a life-threatening complication, should be considered once hemodynamic instability occurs during the procedure.

## 1 Introduction

Thoracentesis is a common invasive procedure performed in various clinical settings. The most common complications include pneumothorax, re-expansion pulmonary edema, vasovagal events, and bleeding.^1^,^2^,^3^ Although organ or artery injury is rare, if it occurs, it usually has lethal consequences.^3^,^4^,^5^,^6^ We describe our experience with a patient, who after left pneumonectomy, experienced acute cardiac tamponade during blind thoracentesis. Timely diagnosis and subsequent pericardiocentesis ultimately saved her life.

## 2 Case presentation

**Figure 1:**
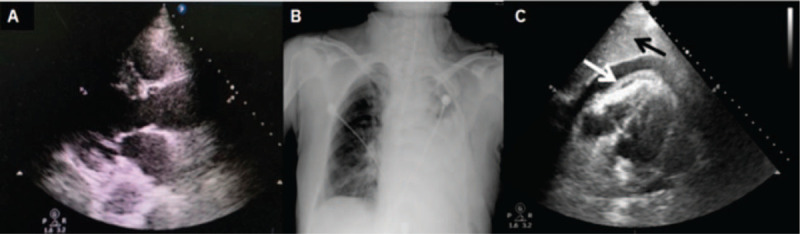
No evidence of pericardial effusion was revealed by transthoracic echocardiography (TTE) before left pneumonectomy surgery (A). Left large pleural effusion was revealed by chest X-ray four days after surgery (B). Large pericardial effusion and suspected right ventricular collapse (white arrow) were revealed by TTE on the subxiphoid four-chamber view, in which the liver (black arrow) was observed to be located between the probe and the heart (C). TTE = transthoracic echocardiography.

A 42-year-old woman with a 20-year history of bronchiectasis was admitted to our hospital with exacerbated hemoptysis and exertional dyspnea. Physical examination revealed dullness to percussion, and breath sounds were not audible on the left side of the chest. Chest computed tomography revealed a destroyed left lung, with visible dilated right middle and lower bronchi. Transthoracic echocardiography (TTE) revealed normal cardiac structure and function (Fig. 1A). She underwent left pneumonectomy surgery 10 days after admission. After surgery, the patient complained of slight breathlessness, with a blood pressure (BP) of 135/88 mm Hg, a heart rate (HR) of 85 beats/min, and a respiration rate of 22 breaths/min. Laboratory investigations revealed a hemoglobin level of 88 g/L (reference, 115–150 g/L), troponin-T of 9 ng/L (reference, 0–14ng/L), and aspartate transaminase of 32 IU/L (reference, < 40 IU/L). Chest radiography revealed large left pleural effusions four days after surgery (Fig. 1B). Blind thoracentesis guided by physical examination was planned.

The patient sat upright, leaning slightly forward with her arms on a chair. A needle was successfully inserted at the 5th intercostal space, in the midaxillary line into the pleural cavity. The patient suddenly lost consciousness after slowly discharging 30 mL of bloody fluid; the procedure was stopped immediately and she was placed supine on a bed. She was diagnosed with shock, with an unmeasurable BP, weak breathing, an HR of 148 beats/min, and an oxygen saturation of 82%. She was incubated immediately and placed on invasive ventilation. Intravenous infusion of epinephrine and vasoactive agents was administered immediately but resulted in poor response. A series of examinations was performed to determine the cause of shock, with TTE revealing pericardial effusion at a depth of 20 mm on subxiphoid view and suspected collapse of the right ventricle during diastole (Fig. 1C). Acute cardiac tamponade was suspected. Pericardiocentesis was performed by an experienced cardiologist, who abandoned a subxiphoid approach due to its high risk for liver injury with needle insertion directed by TTE assessment (Fig. 1C). Ultimately, pericardiocentesis succeeded though an apex approach under TTE guidance. Her BP gradually increased to 123/80 mm Hg and HR decreased to 84 beats/min a few hours after discharge of 250 mL of non-coagulated blood. Laboratory tests revealed that hemoglobin decreased to 64 g/L, troponin-T increased to 604 ng/L, and aspartate transaminase increased to 772 IU/L 7 hours after thoracentesis; myocardial damage was identified. A tube was then placed and retained for the next 36 hours; a total of 330 mL of bloody fluid was drained. The patient was discharged from hospital in a good condition few days later. Written informed consent was obtained from the patient for publication of anonymized case details and any accompanying images.

## 3 Discussion

Although rare, organ damage is the most serious complication of thoracentesis, usually involving the lung(s), liver, or spleen.^[3]^ Cardiac damage, however, is a much rarer complication, with two previous case reports describing fatal consequences. Adeniran et al reported on a 44-year-old woman who died minutes after diagnostic thoracentesis. Autopsy revealed that a large extension of the lung was destroyed by tuberculosis and an enlarged heart (due to hypertensive heart disease) had replaced the space left by the shrunken lung. The cardiac injury was confirmed by evidence of puncture wounds at the apex.^[7]^ Vardi et al described an older woman who died of cardiopulmonary collapse soon after large-bore needle thoracentesis to drain a pleural effusion; mechanical cardiac injury was confirmed by autopsy. Her dilated left ventricle (due to hypertension, coronary heart disease, and chronic renal disease) was close to the left rib cage at the site of the puncture, which was believed to have resulted in fatal consequences.^[8]^ Although the heart of the patient described in the present report was normal, the left pneumonectomy left a space for it to float and was close to the chest wall. Thus, it appears that the altered intra-thoracic structure (ie, destroyed lung or enlarged heart) increased the risk for cardiac injury during thoracentesis. Additionally, clinicians should be acutely aware of such a complication once thoracentesis-related hemodynamic instability occurs. Timely recognition, emergency pericardiocentesis, or surgery are life saving.

Although ultrasound guidance is recommended,^[3]^ the totally excised left lung led the operator to believe that ultrasound guidance was not necessary for the patient in this case to avoid pneumothorax. However, ultrasound can also reveal underlying intra-thoracic abnormalities such as cardiac enlargement or displacement, a raised diaphragm, or adherent lung. It has been reported that ultrasound guidance can prevent potential organ puncture in 10% of procedures.^9^,^10^,^11^ This case further emphasizes the indispensable role of imaging evaluation for thoracentesis, especially in patients with altered intra-thoracic anatomy.

## Author contributions

**Conceptualization:** Jiafu Wei, Mingxia Zheng.

**Methodology:** Mingxia Zheng, Tong Wang.

**Resource:** Jiafu Wei, Tong Wang.

**Writing – original draft:** Yu Kang.

**Writing – review & editing:** Mingxia Zheng.
